# Endogenous Sox8 is a critical factor for timely remyelination and oligodendroglial cell repletion in the cuprizone model

**DOI:** 10.1038/s41598-023-49476-5

**Published:** 2023-12-14

**Authors:** David Freudenstein, Magdalena Lippert, Janina Sophie Popp, Jessica Aprato, Michael Wegner, Elisabeth Sock, Stefanie Haase, Ralf A. Linker, María Nazareth González Alvarado

**Affiliations:** 1 Neuroimmunology Laboratory, Department of Neurology, University Hospital Regensburg, Franz-Josef-Strauss-Allee 11, 93053 Regensburg, Germany; 2 Institute of Biochemistry, Friedrich-Alexander University Erlangen, Erlangen, Germany; 3 Present Address: Institute of Molecular Biotechnology of the Austrian Academy of Sciences (IMBA), Vienna Biocenter (VBC), Vienna, Austria

**Keywords:** Neurological disorders, Myelin biology and repair

## Abstract

Genome-wide association studies identified a single nucleotide polymorphism (SNP) downstream of the transcription factor Sox8, associated with an increased risk of multiple sclerosis (MS). Sox8 is known to influence oligodendrocyte terminal differentiation and is involved in myelin maintenance by mature oligodendrocytes. The possible link of a Sox8 related SNP and MS risk, along with the role of Sox8 in oligodendrocyte physiology prompted us to investigate its relevance during de- and remyelination using the cuprizone model. Sox8^−/−^ mice and wildtype littermates received a cuprizone diet for 5 weeks (wk). Sox8^−/−^ mice showed reduced motor performance and weight compared to wildtype controls. Brains were histologically analysed at the maximum of demyelination (wk 5) and on two time points during remyelination (wk 5.5 and wk 6) for oligodendroglial, astroglial, microglial and myelin markers. We identified reduced proliferation of oligodendrocyte precursor cells at wk 5 as well as reduced numbers of mature oligodendrocytes in Sox8^−/−^ mice at wk 6. Moreover, analysis of myelin markers revealed a delay in remyelination in the Sox8^−/−^ group, demonstrating the potential importance of Sox8 in remyelination processes. Our findings present, for the first time, compelling evidence of a significant role of Sox8 in the context of a disease model.

## Introduction

The proliferation, differentiation and myelination performed by oligodendroglial cells are of paramount importance for enabling rapid saltatory conduction and therefore proper function of the central nervous system (CNS)^1^. Consequently, demyelinating diseases such as MS^2^ or leukodystrophies^3^ result in significant neurological dysfunction and impairment. Proliferation and differentiation of oligodendrocyte precursor cells (OPC) are highly regulated processes that are directly linked to the remyelination capacity of the CNS. Although remyelination is very efficient in animal models, it is highly variable and in part inefficient in human pathophysiology^4^. The exact reason why remyelination fails in the adult human brain remains elusive; yet it is an attractive therapeutic target.

Oligodendrocytes (Ol), as the myelinating cells of the CNS, play a crucial role in remyelination processes in the adult brain. Development of Ol in the CNS is highly dependent on the array of transcription factors of the high-mobility-group sex determining region Y related box (Sox) E family, consisting of Sox8, Sox9 and Sox10. While Sox9 is solely expressed during development, Sox8 and Sox10 are also expressed in adult Ol. Sox9 is fundamental for OPC specification in the murine embryo^5^, whereas Sox10 is essential for Ol differentiation and the induction of myelination^6^. During development, Sox8 seems to provide a certain redundancy to Sox9 and Sox10. Sox8 supports specification of OPC in embryogenesis^7^ as well as terminal differentiation of Ol and myelination by binding to myelin gene promotors^8^. However, the compensatory effects of Sox8 are not sufficient to fully replace the roles of Sox9 and Sox10 as the specification of OPCs and differentiation of Ol are considerably reduced in the absence of Sox9 or Sox10, respectively. Altogether, the vast research on SoxE transcription factors convey that despite showing a high level of redundancy and compensation, each of its members also perform unique functions in the CNS^6,9,10^.

In adult mice, Sox8 seems to have a relevant influence on myelin maintenance in Ol. A mutual compensation has been observed between Sox8 and Sox10. Only the deletion of both transcription factors in mature Ol results in a shivering phenotype, reduction of myelin gene expression, and thinner myelin sheaths^11^.

Interestingly, in genome-wide association studies a SNP downstream of Sox8 was found to be significantly associated with the risk of MS^12,13^. The observed link of a Sox8 associated SNP with the risk of MS prompted us to investigate the relevance of Sox8 in an animal model of toxic demyelination, namely the cuprizone model. This model allows the study of demyelination and subsequent remyelination^14^. Our results indicate that the lack of Sox8 leads to delayed remyelination and failure to replenish the oligodendroglial population. To the best of our knowledge, this is the first report of Sox8 having a relevant impact in the diseased brain. In addition, we report a unique neurological phenotype for Sox8 knockout mice, independently of Sox9 and Sox10, which has not been described before. Overall, this study opens a venue to explore Sox8 as a possible therapeutic target in MS.

## Results

**Sox8**^**-/-**^ **mice** **show** **reduced** **weight** **and** **motor** **performance** **upon** **cuprizone** **diet**.

**Figure 1:**
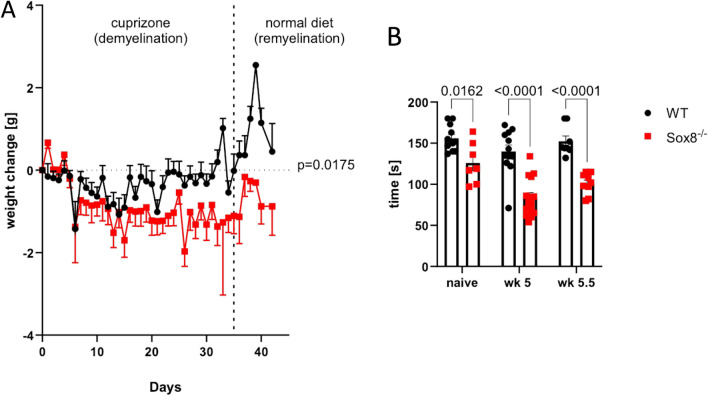
Sox8^−/−^ mice show increased weight loss and impaired motor function upon toxic demyelination. Mice received a 0.2% cuprizone diet for 5 weeks and were fed normal chow for 7 additional days. The dashed line indicates the termination of cuprizone treatment (timepoint of full demyelination and the starting point of remyelination processes). (**A**) Concerning weight change compared to day 0 (start of cuprizone diet), Sox8^−/−^ mice (red) suffered from increased weight loss during cuprizone diet compared to WT controls (black). (**B**) The time of fall from an accelerating rod of Sox8^-/-^ or WT mice before cuprizone treatment (naïve), at the time point of full demyelination (wk 5) and during remyelination (wk 5.5) revealed an increased motor deficit in naïve and cuprizone treated Sox8^−/−^ mice. Bars represent mean, error bars show SEM.

Wildtype (WT) and Sox8^−/−^ mice were fed cuprizone for 5 weeks to induce full demyelination. As expected, body weights decreased due to cuprizone feeding in both groups. Yet, compared to WT controls, Sox8^−/−^ mice displayed a more pronounced decrease in body weight during toxic demyelination (Fig. 1A). Unexpectedly, we observed that the motor skills of Sox8^−/−^ mice differ significantly from the WT littermates even in cuprizone naïve mice (Fig. 1B). The latency of WT littermates to fall of the rotarod did not change significantly due to cuprizone feeding and after termination of the diet. On the contrary, the Sox8^−/−^ group showed a sharp decrease in latency at wk 5 and no recovery during remyelination at wk 5.5 (Fig. 1B).

**Sox8**^**−/**−^ **mice** **presented** **a** **delay** **in** **the** **remyelination** **process**.

**Figure 2:**
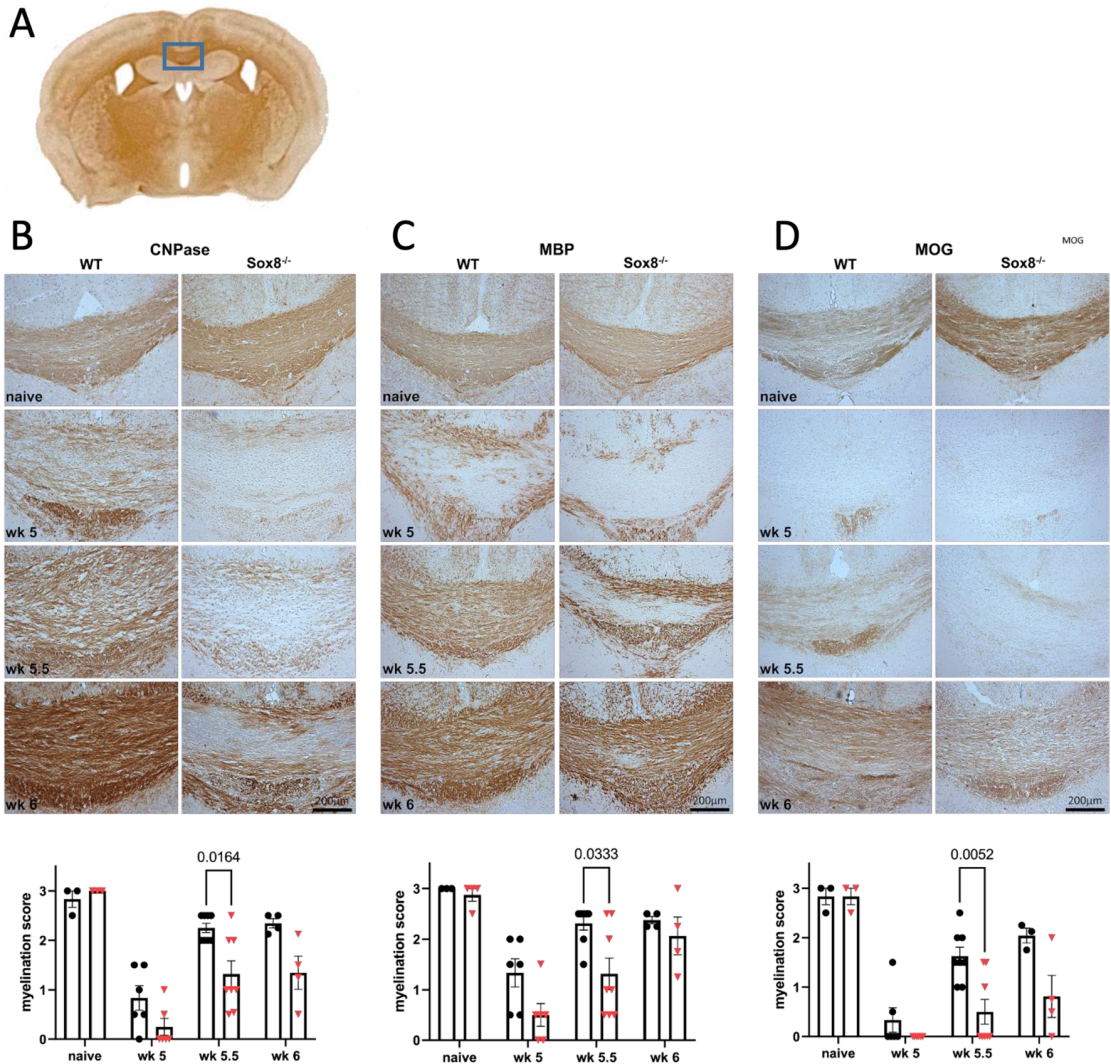
Re-expression of myelin related proteins is delayed in Sox8^−/−^ mice after toxic demyelination. Mice received a 0.2% cuprizone diet for 5 weeks and were fed normal chow for 7 additional days. Myelination was assessed in the median body of the CC, as indicated by the grey rectangle in (**A**). Histochemical staining with anti-CNPase (**B**), anti-MBP (**C**) and anti-MOG (**D**) was performed at full demyelination (wk 5; n = 6 per group) and during remyelination (wk 5.5; n = 8 per group and wk 6; n = 4 per group) in Sox8^−/−^ mice or WT controls compared to naïve mice without cuprizone feeding (n = 3 per group). Representative pictures are shown for each staining and group. Scale bar is 200 μm. Bars represent mean, error bars show SEM.

To evaluate the potential effect of Sox8 on de- and remyelination processes in the cuprizone model, we investigated myelin related proteins CNPase, MBP and MOG in brain sections of Sox8^−/−^ mice and WT controls. Both groups showed similar myelin contents in the median body of the corpus callosum (CC; Fig. 2A) before cuprizone feeding (Fig. 2B–D, naïve). In accordance with the model, following cuprizone diet, there was a notable reduction in the expression of all three myelin proteins at wk 5 in both Sox8^−/−^ and WT mice (Fig. 2). CNPase, MOG and MBP positive areas increased in WT mice after the termination of cuprizone feeding, whereas we observed a delayed increase of expression of all myelin associated proteins during remyelination in Sox8^−/−^ mice at wk 5.5. (Fig. 2). This discrepancy was most pronounced for MOG, which is expressed later in remyelination after cuprizone diet compared to CNPase and MBP^15,16^. By wk 6, the differences between groups were no longer significant, indicating that this might not be a permanent phenotype.

**Oligodendroglial** **cell** **repletion** **after** **toxic** **demyelination** **is** **impaired** **in** **Sox8**^−/−^ **mice**.

**Figure 3:**
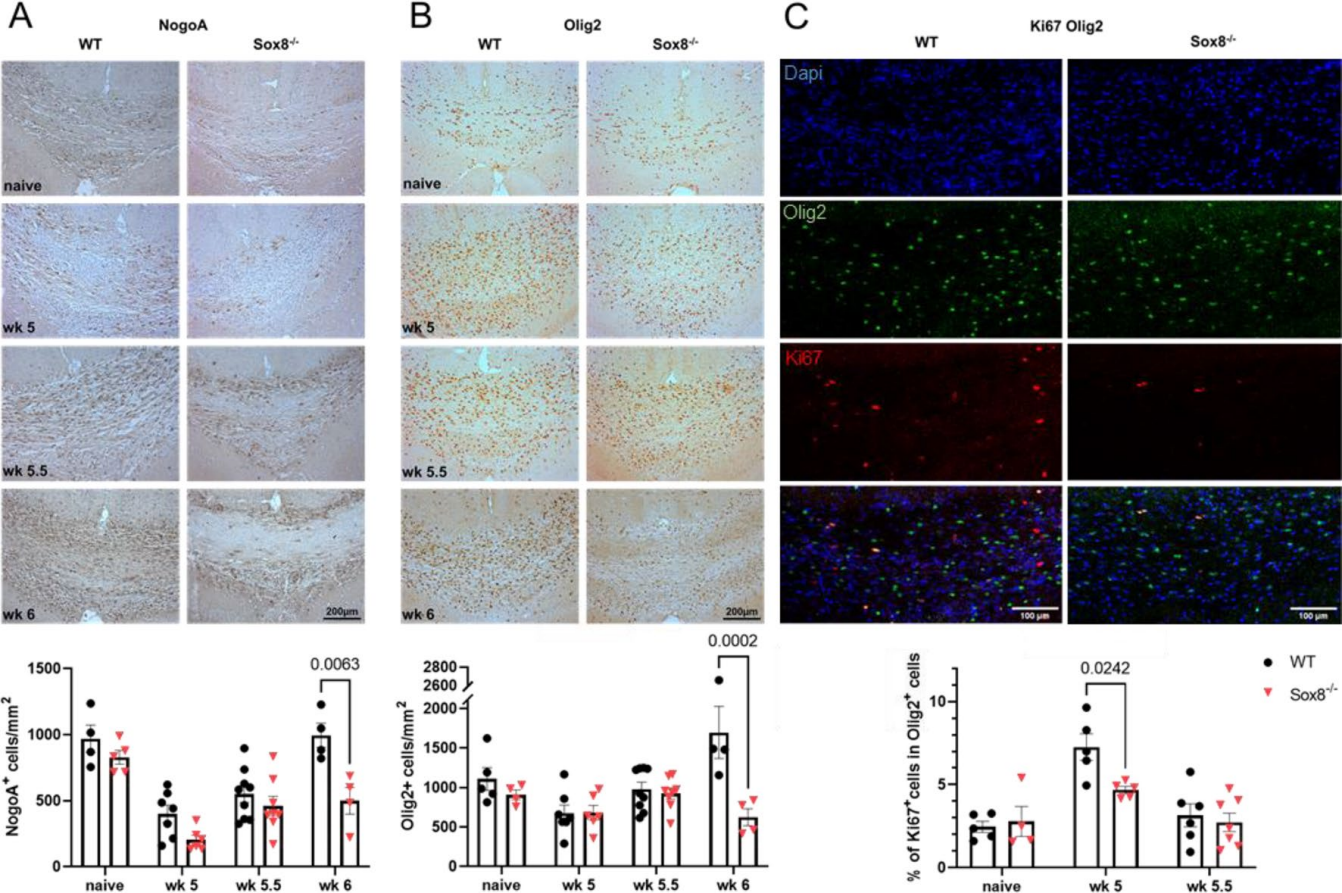
Oligodendroglial cell densities and proliferation are decreased in Sox8^−/−^ mice. Mice received a 0.2% cuprizone diet for 5 weeks and were fed normal chow for 7 additional days. Immunohistochemical staining of the CC for (**A**) NogoA^+^ and (**B**) Olig2^+^ oligodendroglial cells in Sox8^−/−^ mice or WT controls was performed at full demyelination (wk 5; n = 6–7 per group) and during remyelination (wk 5.5; n = 9 per group and wk 6; n = 4 per group) compared to naïve mice without cuprizone feeding (n = 4–5 per group). Representative pictures of the median body of the CC are shown for each staining and group. Scale bar is 200 µm. (**C**) Immunofluorescence staining for Olig2 (green) and Ki67 (red) revealed reduced proliferation of Olig2^+^ cells in Sox8^−/−^ mice at wk 5 (n = 4–7 per group and time point). Exemplary images of Ki67^+^Olig2^+^ cells are shown. Bars represent mean, error bars show SEM.

Since cuprizone-induced loss of myelin is a result of oligodendrocyte apoptosis we performed immunohistochemistry for NogoA^+^ and Olig2^+^ cells to evaluate changes in mature Ol and the overall Ol population. Quantification of NogoA^+^ cells revealed similar densities of mature Ol in naïve Sox8^−/−^ and WT mice in the analyzed area of the CC (Fig. 3A). As expected, after 5 weeks of cuprizone diet, the number of NogoA^+^ Ol was significantly reduced in both groups, with no significant differences between Sox8^−/−^ mice and WT controls. We observed a gradual increase in NogoA^+^ Ol during remyelination. One week after termination of cuprizone diet, densities of mature Ol in WT mice were comparable to the naïve control group. Yet, densities of NogoA^+^ cells failed to replenish in Sox8^−/−^ mice and were significantly reduced as compared to WT controls (Fig. 3A). Similarly, there was no difference in Olig2^+^ cell numbers between Sox8^−/−^ mice and WT controls in the naïve group or at wk 5 of cuprizone diet (Fig. 3B). After the termination of cuprizone feeding, Olig2^+^ cell numbers slightly increased at wk 5.5 compared to wk 5, but there was no difference between Sox8^−/−^ mice and WT controls. However, quantification at wk 6 revealed significantly reduced numbers of Olig2^+^ cells in Sox8^−/−^ mice (Fig. 3B), suggesting gross changes in oligodendroglial cell populations. Since proliferation of OPC is essential for later remyelination, we performed co-staining of Olig2 and the proliferation marker Ki67. Here, we identified significantly decreased numbers of proliferating OPC in Sox8^−/−^ mice at wk 5 compared to WT controls (Fig. 3C).

**Lack** **of** **Sox8** **does** **not** **influence** **the** **level** **of** **gliosis** **in** **the** **cuprizone** **model**.

**Figure 4:**
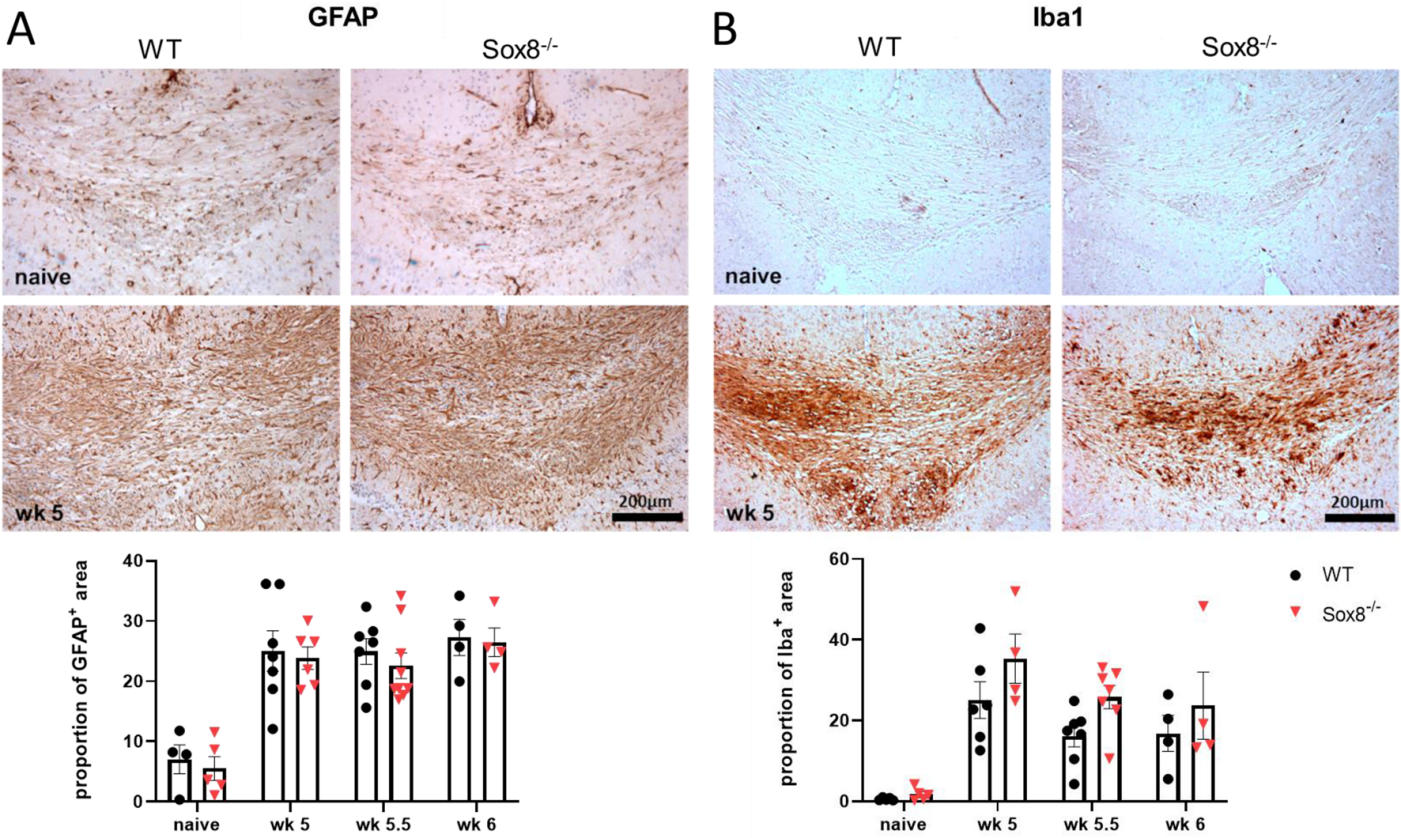
Astrogliosis and microglia activation after toxic demyelination. Mice received a 0.2% cuprizone diet for 5 weeks and were fed normal chow for 7 additional days. Histochemical staining of the CC with (**A**) anti-GFAP and (**B**) anti-Iba1 was performed at full demyelination (wk 5; n = 6–7 per group) and remyelination (wk 5.5; n = 7–9 per group and wk 6; n = 4 per group) in Sox8^−/−^ mice or WT controls compared to naïve mice without cuprizone feeding (n = 4–5 per group). Representative pictures of the median body of the CC are shown for each staining and group. Scale bar is 200 µm. Bars represent mean, error bars show SEM.

To identify a potential impact of Sox8 on gliosis during de- and remyelination, we additionally performed histological analysis of Iba-1 and GFAP expressing cells at wk 5, wk 5.5 and wk 6 in the median area of the body of CC. Analysis of immunopositive areas for either Iba-1 or GFAP indicating microglial or astroglial activation, respectively, showed an expected increase in both glial markers upon demyelination but no obvious differences were found between groups at any time point (Fig. 4).

## Discussion

Mutations linked to transcription factors Sox9 and Sox10 have long been related to diseased states in humans and mice^17,18^. However, much less is known about the role of Sox8 in disease. In 2013 the rs2744148 SNP, located downstream of the Sox8 gene, was associated with a higher risk to develop MS^13^. Moreover, Sox8 is expressed in Ol, the myelinating cells of the CNS, where it influences myelin maintenance and myelin gene expression^11^. Due to its relevance in Ol physiology and connection to MS risk, we examined whether lack of Sox8 may have an effect in the cuprizone model of toxic demyelination.

Sox8^−/−^ mice are viable and do not show any severe developmental phenotype^19^. The most obvious phenotype of these mice lies in smaller fat deposits, that result in ~ 30% lighter weight than WT animals^19,20^. The weight difference between WT and Sox8 deficient mice prior to cuprizone feeding was less pronounced in our experiments, but observable. During the cuprizone diet, we observed that Sox8 deficient mice lose significantly more weight over the course of the cuprizone diet. These results suggest that not only do Sox8^−/−^ mice experience adipose tissue degeneration in adulthood^20^, but they seem to be more prone to weight loss under the cuprizone diet. Whether this tendency to lose weight expands to other stress conditions would need to be evaluated in future studies. In addition, we described a motor deficit in these mice upon cuprizone exposure using the rotarod test^21^. Whether motor skills are diminished in the Sox8^−/−^ line independent of cuprizone induced demyelination in the CC needs to be addressed in future studies using a more nuanced motor behaviour assessment.

Even though we did not assess the muscle tissue in this study, the presence of motor deficits opens an interesting venue of research for Sox8. Previous research has linked Sox8 to myogenic differentiation and it has been recently identified as a genetic variant of interest for severe adolescent idiopathic scoliosis and familial essential tremor^22–24^. Altogether, the behavioral findings of this study further characterized this mouse line and open compelling questions regarding the role of Sox8 in disease.

SoxE transcription factors are closely entangled in Ol physiology. Sox9 is essential for proper Ol specification and Sox10 for terminal differentiation^5,6^. Due to the redundancy with its partners, it has been challenging to identify a unique function for Sox8^7,8^. Only recently, Turnescu et al. have proposed that Sox8 is necessary for myelin maintenance and myelin gene expression^11^. In the present study, we challenged Sox8 deficient mice with the cuprizone model of toxic demyelination which allows for the analysis of both de- and remyelinating stages^14^. This approach may elucidate whether Sox8, by itself, may be relevant in a demyelinating condition. Interestingly, Sox8 deficient mice showed a delay in the remyelinating phase. A similar hiatus has been previously reported in these mice in early developmental stages, where Ol terminal maturation was reduced up to 50% resulting in a transient delay in myelination^8^. However, this mouse line has not been challenged in a disease model before and it is the first time that Sox8 is reported to have an effect in the adult diseased brain.

At week 5, i.e. at maximum of toxic demyelination, Sox8^−/−^ mice showed reduced expression of myelin related genes in the median CC, when compared to WT animals. These findings were not significant, however. Whether maximal toxic demyelination is increased in Sox8^-/-^  compared to WT mice remains uncertain to date.

It is becoming clear that many developmental mechanisms, such as myelination, are to some degree recapitulated during adult regeneration or insult^25–27^. Here, we observe a similar delay in the regenerative process of remyelination as occurs during developmental myelination in the absence of Sox8. To shed light on the cause of the remyelination delay, we analyzed different oligodendroglial sub-populations that are essential for this process. Recent studies have shown that newly generated Ol, derived from OPC, are very efficient during remyelination, in contrast to the modest contribution of surviving mature Ol^28–30^. Hence, we investigated the changes in proliferating OPC during cuprizone. In accordance with the literature^31^, we detected an increase in proliferating glia in CC at the peak of demyelination, an increment that was significantly hindered in the Sox8 deficient group. Additionally, we reported a failure to replenish the mature Ol population during the remyelination stage in Sox8^−/−^ mice. In the cuprizone model, mature Ol numbers decreased dramatically until wk 5. Afterwards, they start to repopulate the demyelinated area until they resemble the original density^31^. Such reduction and eventual recovery of mature oligodendroglia is precisely what we observed in the WT group. The lack of Sox8 might impair OPC proliferation and consequently the proper replacement of mature Ol. They recover slowly in the Sox8^−/−^ group indicating that this failure represents a delay more than a permanent damage, resembling the phenotype observed in Sox8 deficient mice during development. Future studies, implementing either further time points or chronic cuprizone, may help to fully answer this question. While we observed significantly reduced numbers of mature Ol by wk 6, we detected deficits in remyelination in Sox8^−/−^ mice as early as wk 5.5. Therefore, remyelination deficits may not be solely based on a lack of mature Ol, but Sox8 may have a separate effect on remyelination, aside from proliferation and differentiation of oligodendroglial cells.

Local gliosis is characteristic of the cuprizone model, hence we analyzed both astrocytic and microglial response. Sox8 is known to be relevant in the differentiation of astrocytes from neural stem cells, as well as in facilitation of GFAP expression in the developing brain^32^. However, we did not observe significant differences in the level of astrocytic activation as measured by GFAP. The described changes therefore seem to be independent of the local astrogliosis. Microglia rapidly gets activated upon cuprizone diet and it steadily decreases during remyelination^31,33^. Unexpectedly, we observed a tendency for microglia to remain activated in the Sox8 group in early stages of remyelination. In recent years, the ample role that OPC play in neuroinflammation has been recognized, as well as the capacity of OPC to impact other inflammatory cell types, such as microglia^34,35^. OPC have suppressive capacity over microglia and the ablation of this population leads to overactivation of microglia in a neuroinflammatory milieu^35^. It is tempting to speculate that the deficient OPC proliferation observed at wk 5 might be involved in the impaired control of the microglia response. Further studies would be necessary to link the two observed changes.

Altogether, our report enhances the characterization of the Sox8 knockout mouse line, and it highlights its utility to understand the role of Sox8 in disease context. Until now, Sox8 has been given little attention due to the lack of unique functions independently from its partners Sox9 and Sox10. Although Sox8 appears to be dispensable during Ol development, it seems to have greater impact in adult mice and to be specially involved in pathological pathways. In the present study, we showed that lack of Sox8 results in increased motor deficits, weight loss and remyelination delay in the cuprizone model. Additionally, we report a defect in OPC proliferation which may lead to a failure to replenish mature Ol populations and may be possibly involved in deficient microgliosis reduction. In conclusion, our report opens many questions regarding Sox8, not only in MS but other degenerative diseases. The so long shunned member of the SoxE family seems to have more critical functions in pathological conditions than previously considered. It would be of most interest to fully dissect its contributions to disease and analyze its potential as a therapeutic target.

## Material and methods

### Animals

Sox8^+/lacZ^ (Sox8^+/−^) mice on a C57Bl/6 J background^18^ were kept as heterozygotes at the central animal laboratory (ZTL), the animal care facility of the University Hospital in Regensburg (Germany) under a 12 h day/night cycle and standardized environmental conditions. Mice received normal or powder chow and tap water ad libitum. The Sox8 homozygotes were derived from intercrosses of heterozygotes and identified by genotyping as described in Sock et al.^19^. In total, we used 25 wildtype animals (10 female, 15 male) and 24 Sox8^−/−^ animals (10 female, 14 male) and analyzed age and gender matched pairs at each time point. All experiments were approved by the German laws for animal protection and were approved by the local ethic committees for animal welfare (Erlangen 55.2–2532-2–450).

### Cuprizone model

To induce full demyelination in the corpus callosum, 8 to 10 week old Sox8^−/−^ and WT littermates were fed a diet containing 0.2% cuprizone (bis-cyclohexanone-oxaldihydrazone, C9012, Sigma) mixed into a powder standard rodent chow (V1530, SNIFF) for 5 weeks. To study remyelination, cuprizone was removed from the diet after 5 weeks and the mice were placed in fresh cages free of any remnants of the toxin. Animals were then kept for 0.5 and 1 week with normal powder chow. Mice were weighed 3 times per week. Age matched littermates that were not exposed to cuprizone were used as naïve controls.

### Rotarod testing

Rotarod testing was performed as described earlier^36^. Briefly, at wk 4 of the cuprizone diet mice were acclimatized to run on a 30 mm diameter rod rotating with constant speed of 12 rounds per minute (rpm) for 120 s. At the maximum of demyelination (wk 5) and during remyelination (wk 5.5 and wk 6) mice were challenged on the rotarod by increasing the acceleration from 0 to 40 rpm in 180 s. After a rest of 30 min the mice were challenged again in the same way. The latency to fall off the rotarod was measured. This test was also performed in WT and Sox8^−/−^ mice without cuprizone feeding, i.e. the naïve group.

### Histology

Mice were transcardially perfused with 4% paraformaldehyde (PFA, Sigma) at wk 0 (naïve controls), wk 5, wk 5.5 and wk 6. Brains were removed, postfixed for 3–4 h in 4% PFA and paraffin embedded. The tissue was sectioned with a microtome into 5 µm coronal slices. The slides were dewaxed and rehydrated, followed by antigen retrieval by boiling them in 1 mM EDTA (pH 8) for 38 min. After letting the tissue cool down, endogenous peroxidases were blocked with 0.5% H_2_O_2_ in 71% methanol, with 0.2 M NaN_3_ or 3% H_2_O_2_. Afterwards, the slides were blocked with 10% BSA in PBS and incubated overnight with primary antibodies diluted in antibody diluent (DCS) as follows: mouse 2',3'-cyclic nucleotide 3' phosphodiesterase (CNPase) 1:1000 (MAB326R, clone 11-5B, Merck Millipore), mouse glial fibrillary acidic protein (GFAP) 1:500 (644,702, clone 2E1.E9, Biolegend), rat myelin basic protein (MBP) 1:200 (MCA409S, clone 12, BioRad), mouse MBP 1:200 (MAB 682, clone 1, Merck Millipore, use for wk 6), mouse myelin oligodendrocyte glycoprotein (MOG) 1:1000 (MAB5680, clone 8-18C5, Merck Millipore), rabbit neurite outgrowth inhibitor A (NogoA) 1:1000 (AB5888, polyclonal, Merck Millipore), rabbit oligodendrocyte transcription factor 2 (Olig2) 1:500 (AB9610, polyclonal, Merck Millipore), mouse ionized calcium-binding adapter molecule 1 (Iba1) 1:500 (MABN92, 20A12.1, Merck Millipore). After washing, the tissue sections were incubated with 1:200 biotinylated goat anti-mouse IgG (BA9200, Vector Laboratories), goat anti-rabbit IgG (BA1000, Vector laboratories) or rabbit anti rat IgG (BA4001, Vector laboratories) for 45 min at room temperature (RT). For immunodetection the slides were incubated with avidin-coupled peroxidase for 35 min at RT (Vectastain Elite Avidin–Biotin-Complex Kit, Vector laboratories). Di-amino-benzidine (DAB) was used as chromogen and hematoxylin was used as a counterstaining for nuclei visualization.

Immunofluorescence staining of paraffin sections was performed for analysis of proliferative oligodendrocytes. The tissue sections were de-waxed, rehydrated and boiled in 0.1 mM EDTA for 38 min. After washing, the sections were blocked with 10% BSA/PBS for 1 h and kept overnight at 4 °C with mouse Ki67 1:75 (550,609, BD Pharmingen) and rabbit Olig2 1:500 (AB9610 Merck Millipore). After washing the slides, they were incubated with 1:1000 secondary antibody anti-rabbit alexa-fluor 488 (A21425, Thermofisher) and anti-mouse alexa-fluor 555 (A21206, Thermofisher) for 45 min at RT. DAPI was used as a nuclear staining and slides were mounted with antifade progold reagent (P36930, Invitrogen).

### Microscopy and image analysis

Quantification of myelination and glial cells was performed in images of the median area of the body of the CC (compare Fig. 2A) taken by Leica DMR microscope with a 10X magnification (objective lens PL Fluotar 10x/0.3) using the Leica DFC320 camera and the Las X software. Saved images in the TIF format had a resolution of 1.59 pixels per µm and 3.24 megapixels. Sections between Bregma − 0.9 and − 1.6 were selected for quantification (Fig. 2A). Demyelination in the CC was scored according to Lindner et al.^37^ by blinded observers as follows: 0: complete demyelination, 1: 1/3 of corpus callosum myelinated, 2: 2/3 of corpus callosum myelinated, 3: complete myelination. Half points were given when necessary, for statistical evaluation one mean score per animal was calculated from the scores of two independent observers.

Densities of NogoA^+^ and Olig2^+^ cells were assessed by manually counting immunopositive cells with an identified nucleus using the cell counting plugin in imageJ^38^. Densities are given in cells per mm^2^. For GFAP and Iba1 staining, percentage of immunopositive area was determined by defining a color threshold in imageJ to identify positive area.

Images of immunofluorescence stained sections were taken with the Observer Z1 (Zeiss) fluorescent microscope. Saved images in the TIF format had a resolution of 0.78 pixels per µm and 0.69 megapixels. Voxel size was 1.28 µm × 1.28 µm x 1 µm.

### Statistical analysis

For statistical evaluation of weight changes, a mixed effects analysis was performed, for rotarod data, a two-way ANOVA followed by Šidák posthoc test for multiple comparison was employed. For histological data, a two-way ANOVA followed by Tukey’s posthoc test was used. Data are shown as mean ± standard error of the mean (SEM). *P*-values were considered significant at *P* < 0.05.

## Data Availability

All data generated or analyzed during this study are included in this published article.
